# The First *Scube3* Mutant Mouse Line with Pleiotropic Phenotypic Alterations

**DOI:** 10.1534/g3.116.033670

**Published:** 2016-11-04

**Authors:** Helmut Fuchs, Sibylle Sabrautzki, Gerhard K. H. Przemeck, Stefanie Leuchtenberger, Bettina Lorenz-Depiereux, Lore Becker, Birgit Rathkolb, Marion Horsch, Lillian Garrett, Manuela A. Östereicher, Wolfgang Hans, Koichiro Abe, Nobuho Sagawa, Jan Rozman, Ingrid L. Vargas-Panesso, Michael Sandholzer, Thomas S. Lisse, Thure Adler, Juan Antonio Aguilar-Pimentel, Julia Calzada-Wack, Nicole Ehrhard, Ralf Elvert, Christine Gau, Sabine M. Hölter, Katja Micklich, Kristin Moreth, Cornelia Prehn, Oliver Puk, Ildiko Racz, Claudia Stoeger, Alexandra Vernaleken, Dian Michel, Susanne Diener, Thomas Wieland, Jerzy Adamski, Raffi Bekeredjian, Dirk H. Busch, John Favor, Jochen Graw, Martin Klingenspor, Christoph Lengger, Holger Maier, Frauke Neff, Markus Ollert, Tobias Stoeger, Ali Önder Yildirim, Tim M. Strom, Andreas Zimmer, Eckhard Wolf, Wolfgang Wurst, Thomas Klopstock, Johannes Beckers, Valerie Gailus-Durner, Martin Hrabé de Angelis

**Affiliations:** * German Mouse Clinic, Institute of Experimental Genetics, Helmholtz Zentrum München, German Research Center for Environmental Health, 85764 Neuherberg, Germany; † German Center for Diabetes Research, 85764 Neuherberg, Germany; ‡ Research Unit Comparative Medicine, Helmholtz Zentrum München, German Research Center for Environmental Health, 85764 Neuherberg, Germany; § Institute of Human Genetics, Helmholtz Zentrum München, German Research Center for Environmental Health, 85764 Neuherberg, Germany; ** Molecular Animal Breeding and Biotechnology, Gene Center of the Ludwig-Maximilians-Universität München, 81377, Germany; †† Institute of Developmental Genetics, Helmholtz Zentrum München, German Research Center for Environmental Health, 85764 Neuherberg, Germany; ‡‡ Department of Life Science, Tokai University School of Medicine, Isehara, Kanagawa 259-1193, Japan; §§ Department of Neurology, Friedrich-Baur-Institut, Ludwig-Maximilians-Universität München, 80336, Germany; *** German Center for Vertigo and Balance Disorders, Munich, 81377 Germany; ††† Institute of Stem Cell Research, Helmholtz Zentrum München, German Research Center for Environmental Health, 85764 Neuherberg, Germany; ‡‡‡ Institute of Pathology, Helmholtz Zentrum München, German Research Center for Environmental Health, 85764 Neuherberg, Germany; §§§ Cedars-Sinai Medical Genetics Research Institute, Los Angeles, California 90048; **** Institute of Molecular Psychiatry, Medical Faculty, University of Bonn, 53127, Germany; †††† Division of Cardiology, University of Heidelberg, 69120, Germany; ‡‡‡‡ Institute for Medical Microbiology, Immunology and Hygiene, Technische Universität München, 81675, Germany; §§§§ Else Kröner Fresenius Center for Nutritional Medicine, Technische Universität München, 85350 Freising-Weihenstephan, Germany; ***** ZIEL – Institute for Food and Health, Technische Universität München, 85350 Freising-Weihenstephan, Germany; ††††† Department of Dermatology and Allergy, Biederstein, Klinikum Rechts der Isar, Technische Universität München, 80802, Germany; ‡‡‡‡‡ Department of Infection and Immunity, Luxembourg Institute of Health, Esch-sur-Alzette, L-4354 Luxembourg; §§§§§ Department of Dermatology and Allergy Center, Odense Research Center for Anaphylaxis, University of Southern Denmark, DK-5000 Denmark; ****** Institute of Lung Biology and Diseases Member of the German Center for Lung Research (DZL), Helmholtz Zentrum München, German Research Center for Environmental Health, 85764 Neuherberg, Germany; †††††† German Center for Neurodegenerative Diseases, 80336 Munich, Germany; ‡‡‡‡‡‡ Lehrstuhl für Entwicklungsgenetik, Technical University München-Weihenstephan, c/o Helmholtz Zentrum München, 85764 Neuherberg, Germany; §§§§§§ Munich Cluster for Systems Neurology (SyNergy), Adolf-Butenandt-Institut, Ludwig-Maximilians-Universität München, 80336, Germany; ******* Lehrstuhl für Experimentelle Genetik, Technische Universität München, 85350 Freising-Weihenstephan, Germany

**Keywords:** SCUBE3, systemic phenotype, pleitropy, Paget disease of bone (PDB), mouse model

## Abstract

The vertebrate *Scube* (Signal peptide, CUB, and EGF-like domain-containing protein) family consists of three independent members, *Scube1–3*, which encode secreted cell surface-associated membrane glycoproteins. Limited information about the general function of this gene family is available, and their roles during adulthood. Here, we present the first *Scube3* mutant mouse line *(Scube3^N294K/N294K^*), which clearly shows phenotypic alterations by carrying a missense mutation in exon 8, and thus contributes to our understanding of *SCUBE3* functions. We performed a detailed phenotypic characterization in the German Mouse Clinic (GMC). *Scube3^N294K/N294K^* mutants showed morphological abnormalities of the skeleton, alterations of parameters relevant for bone metabolism, changes in renal function, and hearing impairments. These findings correlate with characteristics of the rare metabolic bone disorder Paget disease of bone (PDB), associated with the chromosomal region of human *SCUBE3*. In addition, alterations in energy metabolism, behavior, and neurological functions were detected in *Scube3^N294K/N294K^* mice. The *Scube3^N294K/N294K^* mutant mouse line may serve as a new model for further studying the effect of impaired *SCUBE3* gene function.

The vertebrate *Scube* (Signal peptide, CUB and EGF-like domain-containing protein) family consists of three independent members, *Scube1–3*. These encode secreted cell surface-associated glycoproteins that share a domain, organization of at least five recognizable motifs and the ability to both homo- and heterodimerize (Xavier *et al.* 2013). Human *SCUBE3* was originally identified following transcriptional profiling of vascular endothelial cells and demonstrated significant enrichment in primary osteoblasts and long bones (Wu *et al.* 2004). SCUBE3 is a signal protein that is expressed during embryonic development in several tissues (Xavier *et al.* 2013). In mice, *Scube3* is expressed in ectodermal, endodermal, and mesodermal derivatives, as are other members of the *Scube* gene family (Haworth *et al.* 2007). Expression of these genes has been shown to be dynamic, and both reciprocal and complementary to each other (Xavier *et al.* 2013; Haworth *et al.* 2007).

Although our understanding of the function of *SCUBE3* in embryonic development as well as during adulthood is still marginal, one major role appears to be in bone development and homeostasis, with another one in neurological functions. Interestingly, human *SCUBE3* maps to chromosome 6p21.3, a region that has been linked to Paget disease of bone 1 (PDB1) (Fotino *et al.* 1977; Tilyard *et al.* 1982), which is characterized by focal areas of increased bone turnover (Ralston *et al.* 2008). *SCUBE3* function is also associated with other tissues, for example, *Scube3* overexpression in transgenic mice induced cardiac hypertrophy (Yang *et al.* 2007), and zebrafish Scube3 was recently identified as a key regulator of fast muscle development by modulating fibroblast growth factor signaling (Tu *et al.* 2014). Further associations of Scube3 have been reported with hedgehog signal transduction (Johnson *et al.* 2012), angiogenesis (Yang *et al.* 2013), and the immune system (Luo *et al.* 2012). In addition, deregulation of *SCUBE3* has been found in different tumor tissues such as lung cancer (Wu *et al.* 2011; Zhao *et al.* 2013) or renal carcinomas (Morris *et al.* 2011).

Although SCUBE3 seems to be involved in many different organ systems and diseases, there is no suitable mouse model so far for the study of functional alterations. Recent publications on mice lacking *Scube3* did not show any obvious phenotype (Xavier *et al.* 2010; Xavier *et al.* 2013). In this study, we present the first *Scube3* mutant mouse line with phenotypic alterations: *Scube3^N294K/N294K^*. The mutant mouse line carries a recessive point mutation in *Scube3* and was derived from the Munich *N*-ethyl-*N*-nitrosourea (ENU) mouse mutagenesis project (MEP, Hrabé de Angelis *et al.* 2000; Sabrautzki *et al.* 2012). A systemic phenotypic characterization (Hrabé de Angelis *et al.* 2015) of this new mutant mouse line annotates *Scube3* gene function in mice to bone metabolism and morphology, renal function, and hearing, as well as neurological and behavioral functions and energy metabolism.

## Materials and Methods

### Generation of Scube3^N294K/N294K^ mutants

ENU mutagenesis and breeding were performed as described on a pure C3HeB/FeJ (C3H) background (Hrabé de Angelis *et al.* 2000; Sabrautzki *et al.* 2012; Aigner *et al.* 2011). Briefly, C3H mice were originally purchased from the Jackson Laboratory (Bar Harbor, ME) and ENU (Serva Electrophoresis, Heidelberg, Germany) was applied in three weekly intervals by intraperitoneal injections of 90 mg/kg body weight to 10–12 wk old male mice (G0). G0 mice were mated with wild-type C3H females to produce F1 offspring. F1 males not showing any obvious phenotypic alterations were mated with wild-type C3H females to obtain the G2 generation. We either choose 6–8 female G2 mice for matings with their F1 father or performed intercross matings of G2 mice to produce at least 20 mice (G3 families). Phenotyping for dysmorphological alterations was performed according to a standardized protocol (Fuchs *et al.* 2000). A mutation was confirmed by showing a Mendelian distribution of expected homozygous mutant mice. The *Scube3^N294K/N294K^* mouse line was maintained on the C3H genetic background for more than 10 generations.

### Chromosomal mapping

Homozygous carriers of the G3 generation were mated to C57BL/6J (B6) wild-type mice and the progeny (F1 generation) were intercrossed. DNA was prepared from tail tips of affected offspring (F2 generation). For chromosomal mapping, a microsatellite panel for polymorphic markers between C3H and B6 was used (Hrabé de Angelis *et al.* 2000).

### Whole exome sequencing

For enrichment of exonic sequences, we used the SureSelectXT Mouse All Exon 50 Mb kit (Agilent) followed by Illumina HiSeq2000 sequencing as 100 bp paired-end runs with an average 108 × coverage (> 93% of the target being covered > 20 ×). To search for the causative variants, we compared the sequences of one *Scube3^N294K/N294K^* mouse to one mouse from the C3HeB/FeJ background strain.

### Phenotypic analysis

For the phenotypic characterization of young adult (starting at the age of 7 wk) *Scube3^N294K/N294K^* mutants, a cohort of 15 male and 15 female *Scube3^N294K/N294K^* mutants, as well as 15 male and 15 female wild-type littermate controls (*Scube3*^WT^), was analyzed in the primary phenotyping screen of the GMC (Hrabé de Angelis *et al.* 2015; Gailus-Durner *et al.* 2005; Fuchs *et al.* 2012, 2011). The tests within the phenotyping pipeline and the corresponding age of the animals, as well as references for the protocols, are shown in Supplemental Material, Table S1. For further detailed investigation of observed phenotypes, secondary tests were carried out. Details for the applied protocols are listed below.

### Progression study

Since *Scube3^N294K/N294K^* mice showed signs of an impaired bone metabolism, we tested groups of *Scube3^N294K/N294K^* and *Scube3^WT^* mice at 12, 24, 36, and 52 wk of age for the clinical chemical plasma parameters inorganic calcium (Ca), total inorganic phosphate (P_i_), total alkaline phosphatase (ALP), cholesterol (CHO), triglycerides (TGL), glucose (GLUC), total protein (TP), urea (U), uric acid (UA), and albumin (ALB) by using an AU480 clinical chemistry analyzer (Beckman-Coulter, Krefeld, Germany) and adapted reagent kits provided by Beckman-Coulter. Additionally, pQCT (peripheral Quantitative Computed Tomography, Stratec, Pforzheim, Germany) analyses of the femoral metaphysis and diaphysis were performed at the age of 9 and 12 months. We measured CTX-1 in plasma of 52 wk old mice using RatLaps (carboxy-terminal collagen crosslinks, CTX-1) EIA ELISA from IDS (Frankfurt am Main, Germany) according to the manufacturer’s protocol.

### Analysis of renal function in metabolic cages

A subgroup of 48 animals (12 mutant and control mice each of both sexes) at the age of 34 wk was subjected to a renal function test using metabolic cages for single mice (Tecniplast, Buguggiate, Italy) to collect 48 hr urine samples and monitor water and urine production. During the test, mice had free access to water and pulverized food. Tests were conducted as described previously, starting with a single blood sample collection followed by urine collection over 48 hr (Fuchs *et al.* 2011). Urine and plasma samples were analyzed for a set of 12 clinical chemistry parameters including concentrations of sodium, potassium, and chloride (Na, K, and Cl), Ca, P_i_, creatinine (CREA), U, UA, and GLUC, as well as TP and ALB, as described above.

### Transcriptome analysis

Transcriptome analyses from kidney samples of four *Scube3^N294K/N294K^* and four *Scube3^WT^* male mice at the age of 29 wk were performed following total RNA extraction (RNAeasy Midi kit, QIAGEN). Illumina Mouse Ref8 v2.0 Expression BeadChips were employed as previously described (Horsch *et al.* 2008; Kugler *et al.* 2013). Illumina Genomestudio 2011.1 was used for data normalization (cubic spline) and statistical analysis for the identification of differential gene expression was performed with SAM (Significant Analysis of Microarrays, fold change > 1.6, FDR < 6%) (Saeed *et al.* 2006; Tusher *et al.* 2001). Overrepresented functional annotations were obtained through the use of QIAGEN’s Ingenuity Pathway Analysis (IPA, QIAGEN Redwood City, www.qiagen.com/ingenuity). Expression data are available at the GEO database under GSE56402.

### Inner ear preparation

Inner ears were dissected from the temporal bones of killed animals and fixed in 4% formalin (Carl Roth GmbH, Karlsruhe, Germany). Following dehydration in ethanol, inner ears were immersed in methyl salicylate (Sigma Aldrich, Taufkirchen, Germany) and incubated overnight. Analysis of cleared inner ears was documented by photographic images.

### Feces analysis

Fecal samples were collected in single caged mice for consecutive 5 d. Feces were separated from other material (bedding and food) and desiccated in a drying oven at 60° until weight constancy before bomb calorimetric combustion (IKA C7000, Staufen, Germany). Calorie content of food samples was also determined by bomb calorimetry. Energy uptake was calculated by multiplying food energy content and total amount of food consumed. Egested energy was calculated from feces energy content multiplied by the amount of egested feces (see also Rozman *et al.* 2014).

### Statistical analysis

The Shapiro–Wilk test, as well as histograms and quantile–quantile plots, were used to assess the normality of the investigated parameters. For continuous data meeting the assumption of normality, a two-way ANOVA with a Tukey HSD *post hoc* test or a linear model (including body weight as an additional covariate in some cases) was performed to test genotype–sex interaction effects. In tables, normally distributed data were expressed as means ± SDs. Parameters with a skewed distribution were analyzed using the nonparametric Wilcoxon rank sum test and were presented as median as well as 25th percentile and 75th percentile in the tables. Categorical parameters were analyzed by a Fisher’s exact test and are presented as absolute numbers. For all tests, a *P*-value < 0.05 was used as the level of significance. Since the primary phenotyping screen in the GMC was mainly designed as a high-throughput screen for new phenotypic alterations, a correction for multiple testing of the various parameters was not performed. Data were analyzed using R software (Version 3.0.2; Foundation of Statistical Computing, Vienna, Austria).

Housing and handling of mice was according to the German Animal Welfare Act. The animal experiments were approved by the Government of Upper Bavaria (112-02, 78-06, 144-10, and 126-11).

### Data availability

Data of the complete phenotypic analysis is accessible via the website of the GMC (http://tools.mouseclinic.de/phenomap/jsp/annotation/public/phenomap.jsf), and the *Scube3^N294K/N294K^* mutant mouse line is accessible via the European Mouse Mutant Archive (EMMA, www.infrafrontier.eu) under EMMA ID EM:10872.

## Results

### Generation of Scube3^N294K/N294K^ mice

**Figure 1:**
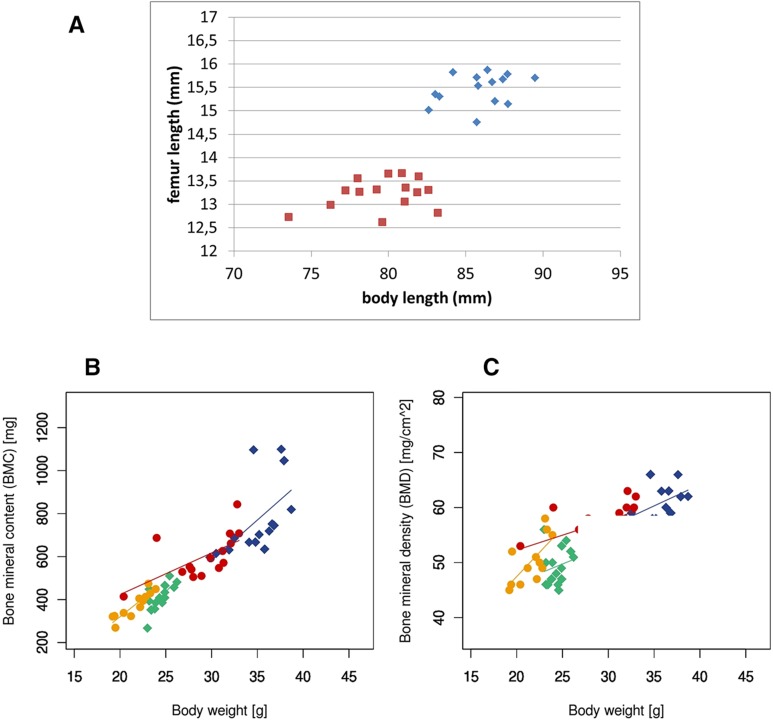
Skeletal abnormalities in *Scube3^N294K/N294K^* mice. (A) Femur length of female animals plotted by body length (red, *Scube3^N294K/N294K^* and blue, *Scube3^WT^*. (B) Bone mineral content plotted by body weight (green, *Scube3^N294K/N294K^* males; yellow, *Scube3^N294K/N294K^* females; blue, *Scube3^WT^* males; and red, *Scube3^WT^* females). (C) Bone mineral content plotted by body weight (green, *Scube3^N294K/N294K^* males; yellow, *Scube3^N294K/N294K^* females; blue, *Scube3^WT^* males; red, *Scube3^WT^* females).

The *Scube3^N294K/N294K^* mutation was generated in the large-scale Munich ENU mutagenesis program. The mutant line was identified by screening G3 animals for morphological abnormalities resulting in reduced body size (Figure 1A), a shorter and kinky tail, abnormal digit positioning, and an abnormal posture when hung by the tail. *Scube3^N294K/N294K^* variants were crossed to wild-type C3HeB/FeJ (C3H) mice, and none of the offspring showed the characteristic *Scube3^N294K/N294K^* phenotypes. However, intercrossing these heterozygous mice resulted in a fraction of about 25% of offspring that showed the characteristic *Scube3^N294K/N294K^* phenotypes. Therefore, we considered *Scube3^N294K/N294K^* to be a recessive mutant mouse line, and maintained the mutation on a pure C3H genetic background for more than 10 generations.

### Mutation detection

Rough mapping by an outcross/intercross breeding strategy with C57BL/6J (B6) animals revealed a 12.4 Mb critical region on chromosome 17 between markers D17Mit46 and D17Mit34, with no recombinants near marker D17Mit198. Whole exome sequencing identified a homozygous nonsynonymous sequence variation within the *Scube3* gene, the only candidate gene within the critical region on chromosome 17. A C–A transversion at nucleotide position 882 in exon 8 of *Scube3* leads to an asparagine to lysine exchange at protein position 294 (N294K). This nonsynonymous sequence variation cosegregated with the *Scube3^N294K/N294K^* phenotype in all 15 tested mutant mice, and was not confirmed in 10 wild-type littermates and other mice of inbred C3H, BALB/c, and B6 strains. Subsequently, this mutation was confirmed in more than 100 mutant *Scube3^N294K/N294K^* mice during maintenance breeding and excluded in the equal number of wild-type littermates.

The N294K substitution is located within the Ca-binding EGF-like domain 7 of SCUBE3, which is highly conserved between mouse, human, zebrafish, and chicken (data not shown). Using the protein prediction programs PROVEAN (http://provean.jcvi.org; Choi *et al.* 2012) and PolyPhen-2 (http://genetics.bwh.harvard.edu/pph2), this sequence variation was classified as deleterious or probably damaging with a score of 0.99.

### Phenotypic analysis

A cohort of 15 male and 15 female *Scube3^N294K/N294K^* mice and the respective number of *Scube3^WT^* mice were analyzed in the GMC in the screens for behavior, neurology, nociception, vision and eye, dysmorphology, bone and cartilage, energy metabolism, hematology, clinical chemistry, steroid metabolism, immunology, allergy, cardiovascular system, lung function, molecular phenotyping, and pathology.

#### Scube3^N294K/N294K^ mice show skeletal abnormalities and changes in bone metabolism:

In addition to the aforementioned smaller body size, shorter and kinked tails, and abnormal digit positioning, X-ray analysis of the skeleton showed no obvious craniofacial abnormalities but detected malformations of the thoracic and lumbar vertebrae, and shorter femora (Figure 1A and Table S2C) in *Scube3^N294K/N294K^* mice that were independent of the body size reduction. Rib fusions occurred with low penetrance (3% of observed animals, data not shown). In DEXA analysis, bone mineral density (BMD) and bone mineral content (BMC) were significantly decreased (Table S2B), but the results might be confounded by body weight (Figure 1, B and C). Several parameters in pQCT-measurements supported the findings of the DEXA analysis. The effects were weaker in 12-month-old than in 9-months-old mice (File S1 and Table S3).

**Figure 2:**
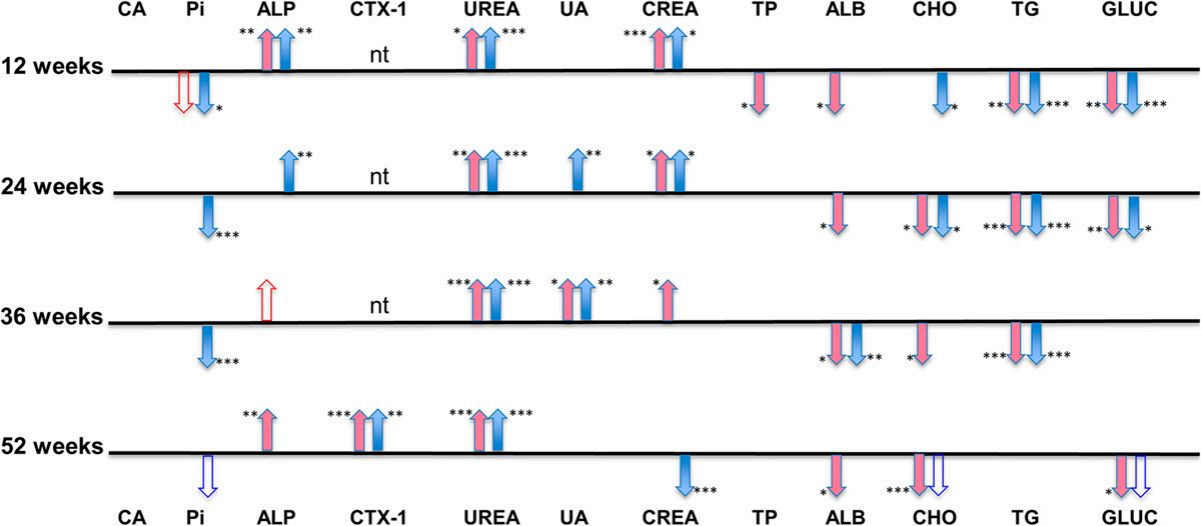
Statistically significantly changed clinical chemical and CTX-1 plasma values of aged groups of *Scube3^N294K/N294K^* mice measured at 12, 24, 36, and 52 wk of age. Red or blue filled arrows show significantly increased or decreased plasma values for female and male mice compared to Scube3^WT^ mice (* *P* ≤ 0.05,** *P* = 0.01,*** *P* ≤ 0.001; nt = not tested). Unshaded but red or blue outlined arrows show a tendency to increased or decreased values not reaching statistically significance. Numbers of mice at 12 wk were female *Scube3^N294K/N294K^* mice *n* = 9, female *Scube3^WT^ n* = 20, male *Scube3^N294K/N294K^* mice *n* = 21, and male *Scube3^WT^ n* = 34; at 24 wk female *Scube3^N294K/N294K^* mice *n* = 10, female *Scube3^WT^ n* = 21, male *Scube3^N294K/N294K^* mice *n* = 15, and male *Scube3^WT^ n* = 29; at 36 wk female *Scube3^N294K/N294K^* mice *n* = 12, female *Scube3^WT^ n* = 8, male *Scube3^N294K/N294K^* mice *n* = 25, and male *Scube3^WT^ n* = 33; and at 52 wk: female *Scube3^N294K/N294K^* mice *n* = 12, female *Scube3^WT^ n* = 8, male *Scube3^N294K/N294K^* mice *n* = 5, and male *Scube3^WT^ n* = 11. For CTX-1 measurement at 52 wk, numbers were *n* = 10 mice for both female *Scube3^N294K/N294K^* mice and *Scube3^WT^* mice, male *Scube3^N294K/N294K^* mice *n* = 9, and male *Scube3^WT^* mice *n* = 10. ALB, albumin; ALP, total alkaline phosphatase; Ca, total inorganic calcium; CHO, cholesterol; CREA, creatinine; CTX-1, carboxy-terminal collagen crosslinks; GLUC, glucose; Pi, total inorganic phosphate; TG triglycerides; TP, total protein; UA, uric acid.

Ca levels at the age of 17 wk were elevated in *Scube3^N294K/N294K^* female mice but this finding was not confirmed in older animals. P_i_ levels were globally decreased in male mice and ALP activities in mice of both sexes were increased with effects getting weaker with age. At 1 yr of age, *Scube3^N294K/N294K^* mice showed a significant increase of the bone resorption marker CTX-1 (Figure 2).

#### Scube3^N294K/N294K^ mice have defects in bone growth, but not in embryonic patterning:

**Figure 3:**
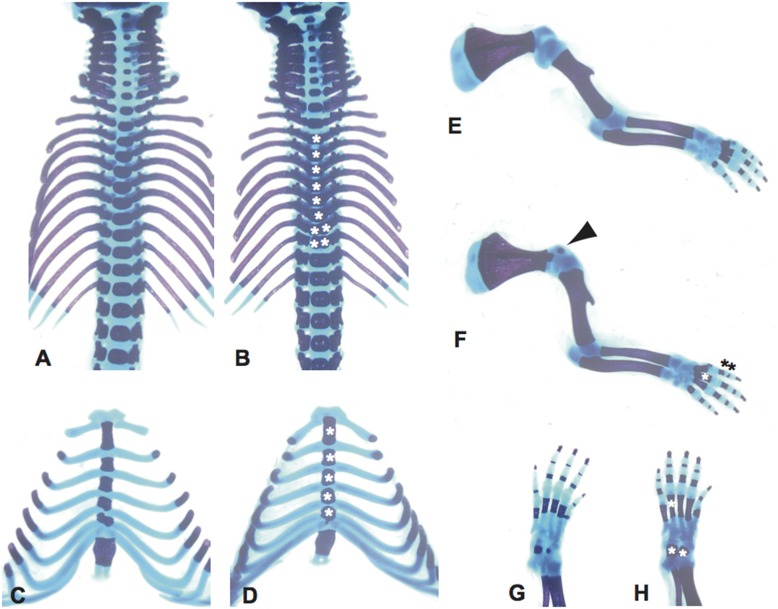
Skeletal phenotypes of newborn *Scube3^N294K/N294K^* mice. Hyper-ossification of entire vertebrae in homozygous *Scube3^N294K/N294K^* mice (B and D) compared to wild-type mice (A and C). Asterisks in (B) indicate remarkable hyper-ossified vertebrae. Note that split ossification centers were detected in T10 and T11 (two asterisks in one vertebra). Sternebrae of *Scube3^N294K/N294K^* mice (D) were slightly thicker and longer than ones of wild-type mice (C). Asterisks in (D) indicate hyper-ossified sternebrae. Bones of upper and lower limbs in homozygous mutants (F and H) were also hyper-ossified compared to wild-type (E and G). Arrowhead indicate ossified coracoid process (E). Asterisks in (F) and (H) show representative hyper-ossified metacarpals, metatarsals, phalanx, and tarsals.

For further investigation of the observed bone abnormalities, we analyzed *Scube3^N294K/N294K^* mice at newborn and various embryonic stages by skeletal staining. Multiple hyper-ossifications in the axial and appendicular skeleton were detected. In thoracic, lumbar, and sacral vertebrae of *Scube3^N294K/N294K^* mice, ossification center and pedicles were more closed and fused compared to those of wild-type mice (Figure 3B, asterisks indicate the ossification center). Further, in some thoracic vertebrae, ossification centers were split in mutant mice (Figure 3B, two asterisks in one vertebrae). In the appendicular skeleton, the coracoid processes showed hyper-ossification, and proximal and middle phalanges in digit II started to be fused (Figure 3F, arrowhead and black asterisks). Furthermore, metacarpal, metatarsal, and tarsal were enlarged due to hyper-ossification (Figure 3F and Figure 3H, white asterisks). These hyper-ossification phenotypes were not detected at embryonic day E15.5 around the time when endochondral ossification starts, but were detected in E17.5 *Scube3^N294K/N294K^* embryos (data not shown), suggesting that malformations of *Scube3^N294K/N294K^* mice are due to defects in bone growth, but not because of disturbed embryonic patterning as in vertebral segmentation disorders.

#### Scube3^N294K/N294K^ mice show symptoms pointing to disturbances in renal function:

U, CREA, and K levels, as well as α-amylase activities, were increased in plasma of *Scube3^N294K/N294K^* mice of both sexes (Table S2B). The findings were consistent for most parameters over the whole life period (measured at 12, 24, 36, and 52 wk of age, Figure 2). In addition, plasma glucose levels, triglyceride, and P_i_ concentrations were decreased in both sexes, whereas total protein, albumin, and cholesterol were decreased only in male mutants (Table S2B). To follow up these findings, we analyzed renal function in metabolic cages. Calculated 24 hr CREA clearance adjusted to body weight was normal in *Scube3^N294K/N294K^* mice, suggesting that a similar amount of plasma was filtered per g of body mass by the kidneys of mutant and control mice within 24 hr (Table 1). However, water uptake and urine production in relation to body mass (Table 1) as well as food consumption (2.85 ± 0.23 *vs.* 2.02 ± 0.35 and 3.18 ± 0.57 *vs.* 2.64 ± 0.47 g/24 hr in mutant *vs.* control males and females, respectively) were significantly higher in *Scube3^N294K/N294K^* mice. Urinary concentrations of electrolytes and U were comparable, while CREA, protein (total protein and albumin), and glucose concentrations were slightly lower, and uric acid level was significantly lower, in *Scube3^N294K/N294K^* mice (data not shown). Consequently, calculated 24 hr excretion values per 25 g body mass and fractional excretion rates were significantly increased in *Scube3^N294K/N294K^* mice for the electrolytes Na, K, Cl, as well as Ca, U, total protein, albumin, and glucose. Uric acid and P_i_ excretion adjusted to body mass was found to be highly variable and did not significantly differ between genotypes (Table 1).

**Table 1:** Renal function analysis

	Males		Females		2-Way ANOVA (*P* Value)		
Parameter	*Scube3^WT^* *n* = 12	*Scube3^N294K/N294K^* *n* = 12	*Scube3^WT^* *n* = 10	*Scube3^N294K/N294K^* *n* = 12	Genotype	Sex	Genotype × Sex
Water uptake/25 g BW (g/24 hr)	3.68 ± 0.81	6.01 ± 1.54	4.65 ± 1.88	6.88 ± 1.53	< 0.001	0.039	0.910
Urine excretion/25 g BW (g/24 hr)	0.59 ± 0.31	1.1 ± 0.43	0.89 ± 0.57	1.20 ± 0.55	0.004	0.201	0.412
Creatinine clearance/25g BW (µg/24 hr)	583 ± 305	548 ± 265	812 ± 564	606 ± 191	0.246	0.168	0.409
Na/25 g BW (µmol/24 hr)	102 ± 50	208 ± 59	181 ± 105	205 ± 102	< 0.001	0.004	0.803
K/25 g BW (µmol/24 hr)	228 ± 109	444 ± 131	392 ± 237	539 ± 192	< 0.001	0.014	0.510
Cl/25 g BW (µmol/24 hr)	125 ± 62	262 ± 71	245 ± 154	364 ± 116	< 0.001	< 0.001	0.758
Ca/25 g BW (µmol/24 hr)	1.2 ± 0.6	2.3 ± 0.8	2.7 ± 1.5	4.1 ± 1.4	< 0.001	< 0.001	0.611
Urea/25 g BW (mg/24 hr)	60 ± 28.6	112 ± 31.9	111 ± 65.3	155 ± 44	0.099	< 0.001	0.671
Total protein/25 g BW (mg/24 hr)	8.2 ± 3.5	11.8 ± 3.3	2.8 ± 1.9	3.7 ± 1.9	0.009	< 0.001	0.107
Albumin/25 g BW (µg/24 hr)	98 ± 39	122 ± 31	108 ± 44	153 ± 50	0.008	0.109	0.411
Glucose/25 g BW (µg/24 hr)	210 ± 90	310 ± 94	427 ± 204	501 ± 121	0.030	< 0.001	0.742
FE Na (%)	0.13 ± 0.05	0.30 ± 0.10	0.18 ± 0.05	0.33 ± 0.08	< 0.001	0.149	0.656
FE K (%)	9.7 ± 3.1	20.4 ± 6.2	13.2 ± 3.3	21.7 ± 4.8	< 0.001	0.082	0.422
FE Cl (%)	0.22 ± 0.08	0.52 ± 0.17	0.31 ± 0.08	0.57 ± 0.13	< 0.001	0.061	0.672
FE Ca (%)	0.10 ± 0.03	0.20 ± 0.07	0.15 ± 0.02	0.28 ± 0.08	< 0.001	< 0.001	0.343
FE urea (%)	19 ± 4.9	34 ± 10.4	28 ± 6.6	41 ± 10.5	< 0.001	0.002	0.708
FE total protein (%)	0.007 ± 0.003	0.012 ± 0.006	0.027 ± 0.009	0.045 ± 0.012	< 0.001	< 0.001	0.016
FE albumin (%)	0.00006 ± 0	0.00009 ± 0	0.00007 ± 0	0.00010 ± 0	< 0.001	0.334	0.596
FE glucose (%)	0.029 ± 0.009	0.043 ± 0.010	0.019 ± 0.007	0.031 ± 0.010	< 0.001	< 0.001	0.720

ANOVA, analysis of variance; Na, sodium; K, potassium; Cl, chloride; Ca, calcium; FE, calculated fractional excretion rate.

#### Transcriptome analysis suggests a role of Scube3 in reabsorption and/or excretion of urine components:

We performed transcriptome analysis of kidney samples from *Scube3^N294K/N294K^* mice. Statistical analysis of gene expression patterns identified 138 differentially expressed genes functionally classified by the following overrepresented terms: cellular development, movement, and proliferation, as well as cardiovascular system function, developmental disorder, and renal and urological disease (Table 2). Additionally, literature-based research revealed several genes associated with expression in proximal tubules (*Havcr1*, *Has2*, *Met*, *Mep1b*, *Mme*, and *Slc29a8*), reabsorption of electrolytes (proximal tubule epithelium: *Cldn10* and *Slc12a1*), and maintenance of salt/water balance of blood (renal medulla: *Acta2*, *Adamts1*, *Ehd3*, *Kcnj15*, and *Umod*). All these genes were down-regulated in kidney.

**Table 2:** Functional classification of regulated genes in kidney

Biological Functions and Disease	Genes	**#** Genes
Cellular development	*Blk*, *Casp3*, *Ccr1*, *Chek1*, *Commd3-Bmi1*, *Cxcl12*, *Eif4g2*, *Ereg*, *Foxc1*, *Gabpa*, *Has2*, *Havcr1*, *Hif1a*, *Hmox1*, *Hoxa10*, *Id4*, *Igf2*, *Jdp2*, *Met*, *Rasgrp1*, *Scel*, *Srpx2*, *Tcf21*, *Tert*, *Tnn*	25
Cellular movement	*Adamts1*, *Arhgap35*, *Ccr1*, *Cxcl12*, *Ereg*, *Foxc1*, *Fpr3*, *Has2*, *Hif1a*, *Hmox1*, *Igf2*, *Ltc4s*, *Mep1b*, *Met*, *Mme*, *Plec*, *Ppt2*, *Rasgrp1*, *Sema6d*, *Slc12a1*, *Srpx2*, *Tgfbr3*, *Tnn*, *Umod*, *Vav3*	25
Cardiovascular system function	*A4gnt*, *Adamts1*, *Casp3*, *Chm*, *Cxcl12*, *Ehd3*, *Foxc1*, *Has2*, *Hif1a*, *Hmox1*, *Hoxa10*, *Ift57*, *Igf2*, *Lefty2*, *Met*, *Plec*, *Srpx2*, *Tcf21*, *Tert*, *Tgfbr3*, *Tnn*, *Trpc1*, *Usp8*, *Vav3*	24
Nervous system function	*Arhgap35*, *Casp3*, *Chm*, *Commd3-Bmi1*, *Cxcl12*, *Fa2h*, *Faim2*, *Gpr37*, *Hif1a*, *Hoxa10*, *Ift57*, *Igf2*, *Madd*, *Met*, *Plec*, *Ppt2*, *Rgs7*, *Sh3gl2*, *Slitrk6*, *Vav3*	20
Cell death and survival	*Adamts1*, *C8orf44-Sgk3/Sgk3*, *Casp3*, *Ccr1*, *Chek1*, *Commd3-Bmi1*, *Cxcl12*, *Fa2h*, *Hif1a*, *Hmox1*, *Id4*, *Igf2*, *Jdp2*, *Met*, *Tert*, *Top1*, *Umod*, *Usp8*	18
Neurological disease	*Casp3*, *Commd3-Bmi1*, *Gnat1*, *Gng7*, *Grik1*, *Hmox1*, *Id4*, *Igf2*, *Kcnip2*, *Man1a1*, *Mdh1*, *Ppp1cb*, *Ppt2*, *Ranbp1*, *Sgtb*, *Sh3gl2*, *Top1*	17
Cancer	*Acta2*, *Bdh1*, *Gpr37*, *Havcr1*, *Hif1a*, *Hmox1*, *Id4*, *Igf2*, *Lefty2*, *Mdh1*, *Met*, *Tert*, *Tgfbr3*	13
Cell cycle	*Chek1*, *Commd3-Bmi1*, *Has2*, *Havcr1*, *Hmox1*, *Hoxa10*, *Id4*, *Igf2*, *Met*, *Slbp*, *Tert*, *Trpc1*, *Usp8*	13
Cellular growth and proliferation	*Chek1*, *Cxcl12*, *Ereg*, *Ghrhr*, *Has2*, *Hif1a*, *Hoxa10*, *Id4*, *Lmo3*, *Met*, *Tert*, *Top1*	12
Developmental disorder	*Chm*, *Commd3-Bmi1*, *Eif4g2*, *Has2*, *Hif1a*, *Hmox1*, *Ift57*, *Igf2*, *Ranbp1*, *Sh3gl2*, *Slc12a1*, *Usp8*	12
Renal and urological disease	*Ccr1*, *Ereg*, *Havcr1*, *Hif1a*, *Igf2*, *Lmo3*, *Met*, *Mme*, *Slc39a8*, *Tert*	10

Shown are significantly (*P* < 0.05) enriched terms from the “biological functions and disease” analysis in Ingenuity Pathway Analysis.

#### Scube3^N294K/N294K^ mice have hearing deficits, alterations in the inner and middle ear, and show behavioral abnormalities:

**Figure 4:**
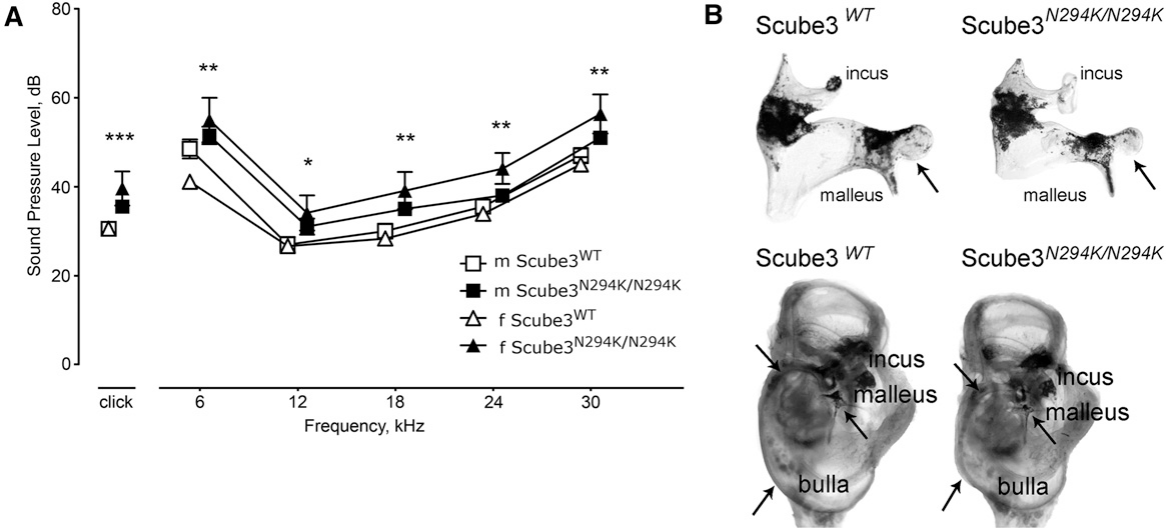
(A) Mild conductive hearing loss; asterisks mark genotype effect of both sexes together. * *P* < 0.05, ** *P* < 0.01, *** *P* < 0.001. (B) Abnormalities in inner ear development. Upper panel: depiction of smaller and malformed ossicles. The ambos (incus) has a different shape and the body of the hammer (malleus) is narrowed (arrow) and the head surface decreased. Lower panel: the whole inner and middle ear was reduced in size and, besides the alterations of the ossicles (arrow pointing toward the hammer, malleus), the auditory cavity (bulla) has an irregular shape (arrows). WT, wild-type.

Hearing sensitivity was assessed by auditory brainstem response (ABR) to different auditory stimuli. There were significant differences at clicks and all tested frequencies; thresholds were increased in *Scube3^N294K/N294K^* mice of both sexes, with female *Scube3^N294K/N294K^* mice more severely affected (Figure 4A). Further analysis of this finding revealed alterations in inner and middle ear preparations. The inner and middle ears appeared smaller and the ossicles of the middle ear had decreased size and altered shape. The incus was smaller, and the body and head of the malleus were especially decreased in size (Figure 4B). The middle ear cavity, the bulla, part of the temporal bone, had an irregular shape as well (Figure 4B).

**Figure 5:**
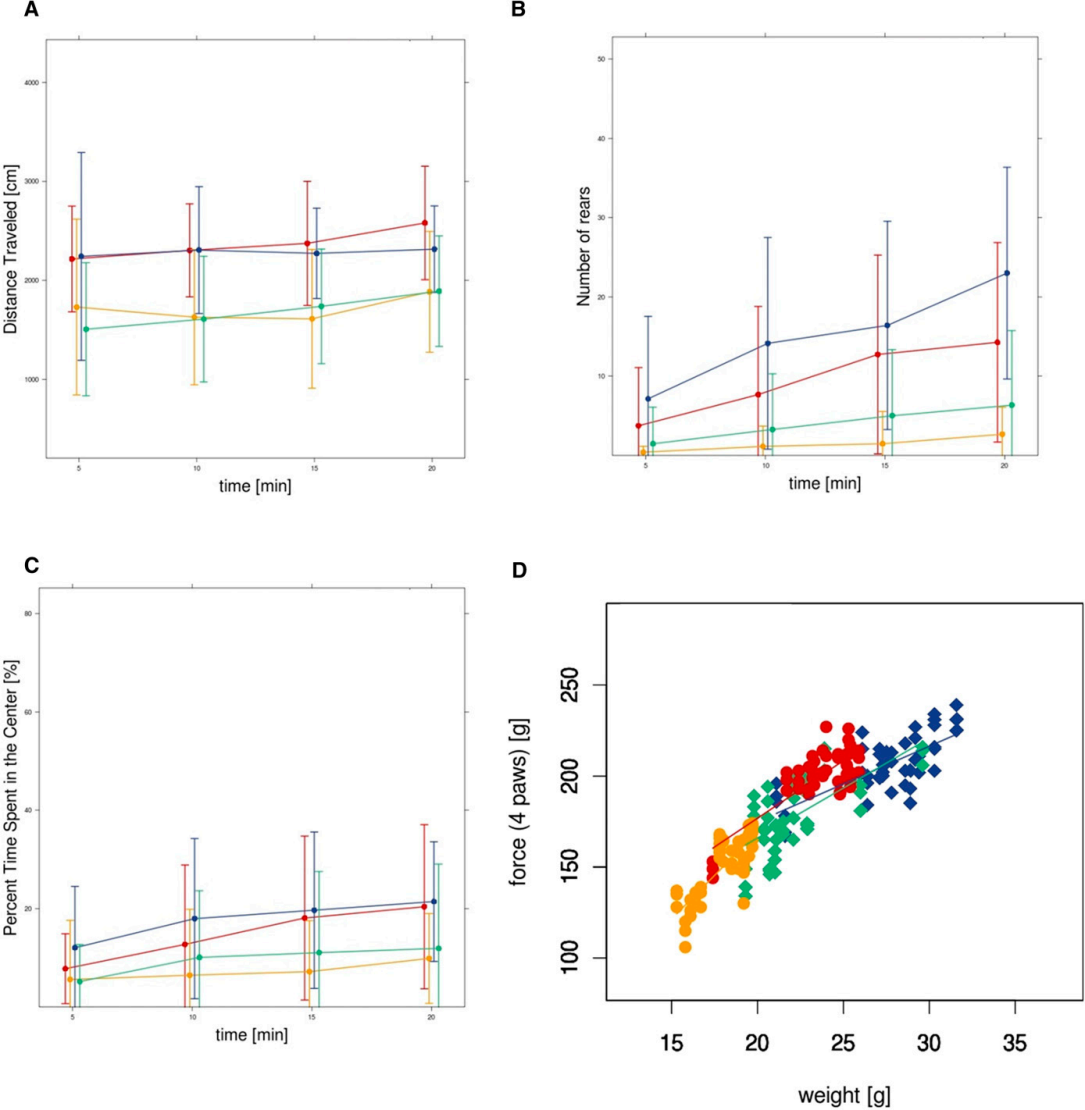
Analysis of *Scube3^N294K/N294K^* mice in the Open Field test and grip strength analysis. Data for distance traveled (A), number of rears (B), and percent time spent in center (C) are shown over the 20 min test interval. Grip strength (four paw measurement) is plotted *vs.* body weight (D) (green, *Scube3^N294K/N294K^* males; yellow, *Scube3^N294K/N294K^* females; blue, *Scube3^WT^* males; and red, *Scube3^WT^* females).

In the open field test, *Scube3^N294K/N294K^* mice showed clearly decreased locomotor and exploratory activity, as indexed by a reduction in total distance traveled and rearing activity over the course of the 20 min test. Movement velocity was also decreased. In terms of anxiety-related behavior, *Scube3^N294K/N294K^* mice spent less time in the center, entered into the center less often, traveled less distance within this zone, and exhibited an increased latency to enter the center (Figure 5 and Table S2B). In the modified hole-board test, maximum and angular velocity were decreased in *Scube3^N294K/N294K^* mice as well as the latency to engage in risk assessment by the male mutant mice (Table S2B).

In a general observation series according to the modified SHIRPA protocol, we also observed reduced locomotor activity, particularly in female *Scube3^N294K/N294K^* mice. In addition, we found a reduction in grip strength, which was also influenced by the body mass reduction (Table S2, B and C and Figure 5).

#### Scube3^N294K/N294K^ mice have altered function in energy metabolism:

**Figure 6:**
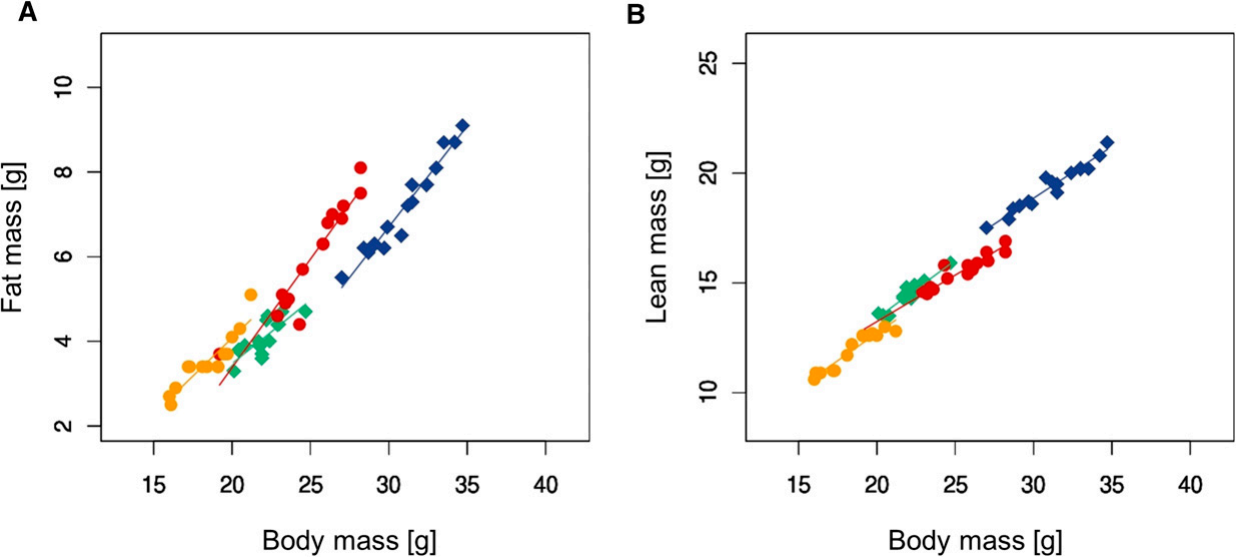
(A) Fat mass plotted against body mass (green, *Scube3^N294K/N294K^* males; yellow, *Scube3^N294K/N294K^* females; blue, *Scube3^WT^* males; and red, *Scube3^WT^* females). (B) Lean mass plotted against body mass (green, *Scube3^N294K/N294K^* males; yellow, *Scube3^N294K/N294K^* females; blue, *Scube3^WT^* males; and red, *Scube3^WT^* females).

*Scube3^N294K/N294K^* mice had significantly reduced body mass, and considerably lower absolute fat and lean mass (Table S2C). They also showed a significant shift in body composition, particularly with regards to decreased lean mass when adjusted to body mass (Figure 6). Interestingly, the relations between body mass and fat content, as well as body mass and lean mass, were significantly different between genotypes (Table S2C), indicating a systematic effect on body composition. *Scube3^N294K/N294K^* mice had significantly lower glucose, triglyceride, NEFA, and glycerol concentrations in plasma (Table S2B). In the glucose tolerance test, basal fasting glucose levels were significantly decreased in *Scube3^N294K/N294K^* mice. AUC (area under the curve) values were decreased, which indicates an improved glucose tolerance in *Scube3^N294K/N294K^* mice (Table S2B). We applied indirect calorimetry to investigate energy expenditure, substrate use, and locomotor activity under home-cage conditions, and found rearing behavior decreased during the 21 hr test phase. No major effects on energy turnover could be detected when VO2 was adjusted to body mass (Table S2C). In a separate test, food consumption and gastro-intestinal functions of *Scube3^N294K/N294K^* mice were monitored over 5 d in single caged animals. We could not detect considerable effects on food intake, energy assimilation, and the efficiency of energy extraction from food.

#### Scube3^N294K/N294K^ mice show mild alterations in cardiovascular parameters, but no hints of deficits in heart performance or conduction:

Echocardiography and electrocardiography revealed several mild alterations in *Scube3^N294K/N294K^* mice (decreased interventricular septum width, decreased left ventricular posterior wall thickness, decreased left ventricular mass, and prolonged QRS interval duration mainly in male *Scube3^N294K/N294K^* mice, as well as decreased diastolic ventricular dimension and decreased respiration rate in female *Scube3^N294K/N294K^* mice, Table S2A). We found a significantly lower heart weight of *Scube3^N294K/N294K^* mice as compared to sex-matched controls (also when normalized to tibia length). However, no alterations were found in heart performance or conduction, and thus there was no clear hint for a physiologically relevant constraint of cardiovascular function due to the mutation. Further in line, the observed alterations may be confounded by the reduced body weight of *Scube3^N294K/N294K^* mice.

#### Further tested organ functions are normal in Scube3^N294K/N294K^ mice:

The analysis of immunologically relevant parameters revealed no major changes. We observed tendencies toward decreased frequency of L-selectin expressing cells within T cell populations and increased frequency of CD11b-expressing cells within the NK cell compartment (Table S2A). We did not detect any changes in hematological parameters. Eye morphology and vision were as expected for the genetic background (C3HeB/FeJ). There were no major genotype effects on prepulse inhibition response or pain perception. No differences were detected for rotarod performance. The analysis of transepidermal water loss (TEWL) from the skin of the mice did not reveal genotype-specific differences, while hair structure and coat appeared normal. There were no alterations in lung function. *Scube3^N294K/N294K^* mice at the age of 21 wk did not present additional histopathological alterations.

## Discussion

**Figure 7:**
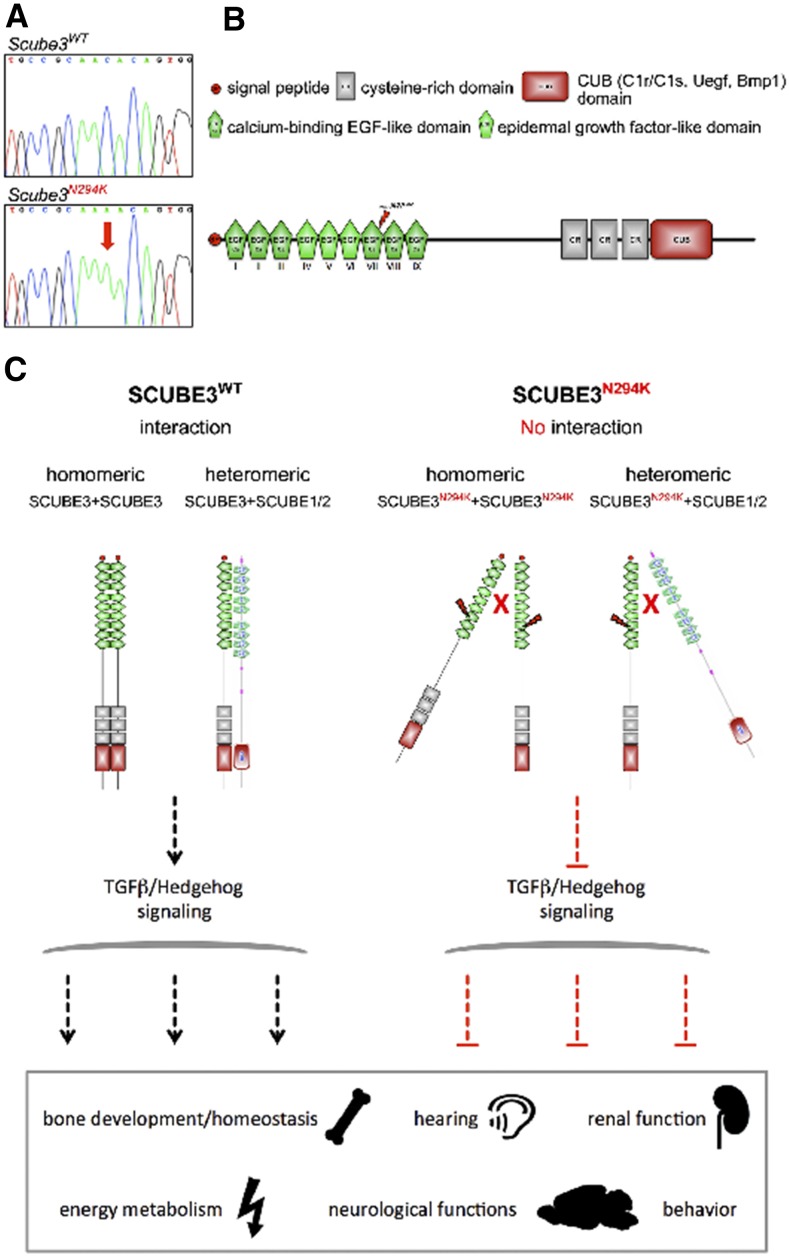
*Scube3^N294K/N294K^* mice are homozygous for a (C) to (A) point mutation at nucleotide position 882 that leads to an asparagine to lysine exchange at protein position 294 (N294K) in exon 8 of *Scube3* (A). The mutation affects the calcium-binding EGF-like domain VII (B), which might have effects on the capabilities to form homo- or heterodimers and block TGFβ/Hedgehog signaling (C), which causes phenotypic alterations in bone development and homeostasis, hearing ability, renal function, energy metabolism, neurological functions, and behavior. EGF, epidermal growth factor; TGFβ, transforming growth factor β.

We identified an ENU-derived mouse line carrying a missense mutation in *Scube3* (NM_001004366.1:c.882C > A, NP_001004366.1:p.Asn294Lys), and characterized the *Scube3^N294K/N294K^* mutant mouse in detail in the GMC (Hrabé de Angelis *et al.* 2015; Gailus-Durner *et al.* 2005; Fuchs *et al.* 2012). As shown previously in a *Scube3* reporter mouse line, targeted replacement of exons 2 and 3 by a *lacZ*-cassette demonstrated early expression of the gene in craniofacial, limb, and neural tube tissues (Xavier *et al.* 2010). However, these mice, as well as SCUBE3 loss-of-function mice, developed normally and did not show overt phenotypic alterations (Xavier *et al.* 2013, 2010) whereas *Scube3^N294K/N294K^* mice exhibited alterations in several organ systems. One reason for this discrepancy might be owed to the different genetic backgrounds of the mice in these studies. However, a more likely explanation is the redundancy of *Scube* family members, which might compensate the loss of SCUBE3 in the null mutant (Xavier *et al.* 2013), and the nature of the mutation in the *Scube3* mouse model described here. The amino acid exchange in *Scube3^N294K/N294K^* mice lies within the EGF-like domain 7 that follows the consensus sequence D/N-X-D/N-E/Q-X_m_-D/N*-X_n_-Y/F for Ca-binding EGF-like domains (cbEGFs, Figure 7). These domains are known to mediate protein–protein and protein–carbohydrate interactions in a Ca-dependent manner (Downing *et al.* 1996). They are also structurally important for several cellular processes such as extracellular matrix architecture or specification of cell fates (Downing *et al.* 1996). For example, missense mutation of conserved amino acids in cbEGFs of fibrillin 1 (FBN1) are causative for Marfan syndrome due to Ca^2+^-dependent misfolding of the protein (Whiteman *et al.* 2007). Likewise, the amino acid exchange in the cbEGF domain 7 in *Scube3^N294K/N294K^* mice might lead to a reduced Ca^2+^-binding capacity and/or to conformational changes, which in turn could negatively interfere with both homo- and heteromeric interaction of SCUBE proteins (Wu *et al.* 2004; Yang *et al.* 2002), posttranslational processing (Wu *et al.* 2011), and interaction with transforming growth factor-β and hedgehog signaling (Johnson *et al.* 2012; Wu *et al.* 2011).

*Scube3* is expressed in the cartilaginous primordia of the skeleton and regions of intramembranous bone formation in the developing craniofacial region (Haworth *et al.* 2007). Nevertheless, besides middle ear abnormalities, *Scube3^N294K/N294K^* mice did not show any further craniofacial abnormalities, suggesting that SCUBE3 is dispensable for craniofacial development (Xavier *et al.* 2013) or that the respective functions may be assigned to regions upstream of cbEGF7 of SCUBE3. However, the *Scube3^N294K/N294K^* mutation directly affects bone morphology and bone metabolism. *Scube3^N294K/N294K^* mice had a significant reduction in body size and weight. Interestingly, GWAS found *SCUBE3* SNPs among adult and pediatric height-associated loci (Gudbjartsson *et al.* 2008; Zhao *et al.* 2010), as well as in pigs, associated with body height, body length, and rump circumference (Wang *et al.* 2014). The influence of the *Scube3* mutation on bone metabolism was expressed by increased ALP activities and CTX-1 values in plasma, as well as by decreased BMD and BMC in DEXA and pQCT analysis. These data are supported by the finding that SCUBE3 is expressed in early osteoblasts and long bones (Wu *et al.* 2004). Interestingly, the human *SCUBE3* gene is located on chromosome 6p21.3, which was discussed to be associated with Paget disease of bone 1 (PDB1) (Wu *et al.* 2004; Fotino *et al.* 1977; Tilyard *et al.* 1982; Good *et al.* 2002). Although PDB is characterized by late onset and mostly focal bone abnormalities due to increased bone turnover (Ralston *et al.* 2008), mouse models for genes known to be mutated in PDB, such as *Tnfrsf11a* for PDB2 (OMIM 603499) and *Sqstm1* for PDB3 (OMIM 601530), have bone abnormalities already at birth (Dougall *et al.* 1999; Li *et al.* 2000) or not until adulthood (Kapur *et al.* 2004; Durán *et al.* 2004; Daroszewska *et al.* 2011). Interestingly, cases of PDB were described to be associated with raised ALP activities and deafness (Tan *et al.* 2014). Therefore, we suggest *Scube3^N294K/N294K^* mice as a new model for further studies on PDB1.

*SCUBE3* maps to a region that was associated with progressive bilateral hearing loss of the mid and high frequencies (DFNA31, OMIM %608645) (Snoeckx *et al.* 2004). The results of the ABR-hearing assessment and the morphological analysis of the middle ears of *Scube3^N294K/N294K^* animals indicate conductive hearing loss. In *Scube3^N294K/N294K^* mice, hearing loss might be a consequence of retarded development of middle ear cavity and ossicle malformations. By contrast, Xavier *et al.* (2013) did not observe any gross abnormalities in the (inner) ears of *Scube3*^−/−^ mice at the age of E17.5. Since background-specific genetic modifiers play an important role in the development of inner ears (Kiernan *et al.* 2007), the differences in the genetic background could be the reason for the different observations in the two studies.

The kidneys play a central role in the regulation of mineral homeostasis and chronic kidney disease (CKD) is associated with bone metabolic disease. However, the regulatory pathways involved are still not fully understood (reviewed in Hu *et al.* 2013; Peacock 2010; Razzaque 2011; Rowe 2012). Elevated plasma U and CREA levels found in *Scube3^N294K/N294K^* mice hint toward possible effects on renal function. We found increased electrolyte and water excretion, but no alteration of CREA clearance. These findings could be a sign of altered renal tubular function or might be a consequence of increased electrolyte consumption due to higher food uptake causing an upregulation of excretory mechanisms. The annotations of differentially expressed genes in kidney with reabsorption of electrolytes and salt/water balance indicate effects on tubular reabsorption. *Acta2* (Tanabe *et al.* 2012), *Adamts1* (Schrimpf *et al.* 2012), *Ehd3* (George *et al.* 2011), *Kcnj15* (Derst *et al.* 1998), and *Umod* (Rampoldi *et al.* 2011) are annotated with expression in renal medulla and salt/water balance, and *Clnd10* (Krug *et al.* 2012), *Havcr1* (Adiyanti and Loho 2012), *Has2* (Michael *et al.* 2011), *Mep1b* (Oneda *et al.* 2008), *Met* (Zhou *et al.* 2013), *Mme* (Van der Hauwaert *et al.* 2013), Slc12a1 (Yang *et al.* 1996), and *Slc39a8* (Nebert *et al.* 2012) are associated with electrolyte reabsorption in the proximal tubules. Together these findings hint toward a possible role of *Scube3* in kidney development and function, supported by the expression of *Scube3* in the developing kidney (Haworth *et al.* 2007), or might be related to secondary effects on the regulation of kidney function.

The human SCUBE3 protein is involved in hedgehog and TGF-β1 signaling (Xavier *et al.* 2013; Haworth *et al.* 2007; Yang *et al.* 2007; Johnson *et al.* 2012) but, so far, nothing is known about SCUBE3 signaling in this network in mice. Transcriptome profiling analysis of kidney from *Scube3^N294K/N294K^* mice identified potential down-stream targets of SCUBE3 that are annotated to TGF-β1 signaling [*C1qtnf3* (Hofmann *et al.* 2011), *Foxc1* (Xu *et al.* 2012), *Has2* (Davies *et al.* 2005), *Jdp2* (Mito *et al.* 2013), *Kcnip2* (Kaur *et al.* 2013), *Lefty2* (Saijoh *et al.* 2000; Arganaraz *et al.* 2012), *Tgfbr3* (Walker *et al.* 2011), and *Tnn* (Alejandre Alcázar *et al.* 2011)]. Reduced expression of all these genes gives evidence for a supportive role of SCUBE3 within the TGF-β1 signaling pathway by binding to transforming growth factor β receptor 2 (TGFBR2) (Yang *et al.* 2007).

Although we did not observe structural defects of muscle tissue in the pathological examination, the repeated findings of reduced locomotor and rearing activity as well as movement velocity might be indications for functional defects, which might also be associated with an abnormal muscle energy metabolism. Indeed, very recent findings from detailed analysis of zebrafish *scube3* suggest that its gene function is crucial for fast-fiber myogenesis (Tu *et al.* 2014). Still, decreased activity and movement velocity could also be due to the described early developmental expression of *Scube3* in the neural tube (Haworth *et al.* 2007).

*SCUBE3* expression was observed in human cultured coronary smooth muscle cells and at low levels in the heart (Wu *et al.* 2004), and its overexpression led to cardiac hypertrophy in transgenic mice (Yang *et al.* 2007). Whereas young *Scube3* transgenic mice appeared phenotypically normal but showed abnormal repolarization ECG patterns as well as thickening of left-ventricular septum and posterior wall thickness when analyzed at 8 months of age (Yang *et al.* 2007), *Scube3^N294K/N294K^* mice showed only very mild alterations in cardiac parameters of our phenotypic analysis. Possibly, they were too young for manifestation of a severe cardiac dysfunction.

In this study, we characterized the first *Scube3* mutant mouse line with phenotypic alterations, suggesting a role of *Scube3* in bone metabolism and morphology, hearing, and renal function. The observed morphological abnormalities of the skeleton, impaired bone metabolism, and hearing impairments correlate with the rare metabolic bone disorder PDB, associated with the chromosomal region of human SCUBE3. Further phenotypic alterations were observed in energy metabolism parameters, in behavior, and neurological functions (Figure 7). This new mouse model will help us to better understand SCUBE3 function and diseases related to *Scube3* gene mutations.

## Supplementary Material

Supplemental Material
