# Supplementary material for: The First Scube3 Mutant Mouse Line with Pleiotropic Phenotypic Alterations
Source: G3 (Bethesda). 2016 Nov 4;6(12):4035–46. doi: 10.1534/g3.116.033670 (PMC5144972; doi:10.1534/g3.116.033670)
Supplement: Supplemental Material [file supp_6_12_4035__index.html]

The First Scube3 Mutant Mouse Line with Pleiotropic Phenotypic Alterations — Supplemental Material 

# The First *Scube3* Mutant Mouse Line with Pleiotropic Phenotypic Alterations

## Supplemental Material for Fuchs, *et al*, 2016

**Files in this Data Supplement:**

- File S1 - Supplemental references. (.pdf, 34 KB)
- Table S1 - Applied phenotyping pipeline in the German Mouse Clinic for the primary phenotype assessment of 15 male and 15 female homozygous *Scube3N294K/-* mutants in comparison to 15 male and 15 female littermate wild-type controls. (.pdf, 23 KB)
- Table S2 - Parameters that showed relevant and significant changes in primary GMC phenotypic analysis pipeline. (.pdf, 162 KB)
- Table S3 - pQCT analysis of 9 and 12 months old Scube3N294K/- mice (.pdf, 129 KB)
