# Supplementary material for: The First Scube3 Mutant Mouse Line with Pleiotropic Phenotypic Alterations
Source: G3 (Bethesda). 2016 Nov 4;6(12):4035–46. doi: 10.1534/g3.116.033670 (PMC5144972; doi:10.1534/g3.116.033670)
Supplement: Supplemental Material [file supp_g3.116.033670_FileS1.pdf]

Supplemental References

Fulton, R. J., R. L. McDade, P. l. Smith, L. J. Kienker, and J. R. Kettman, 1997 Advanced multiplexed analysis with the flowmetrix system. Clin. Chem.,43,1749–1756.

Fuchs H., K. Schughart, E. Wolf, R. Balling, and M. Hrabé de Angelis, 2000 Screening for dysmorphological abnormalities--a powerful tool to isolate new mouse mutants. Mamm. Genome, 11, 528-530.

Fuchs H., T. Lisse, K. Abe and M. Hrabe de Angelis, 2006 Screening for bone and cartilage phenotypes in mice. In. Phenotyping of the Laboratory Mouse. Eds.: Hrabe de Angelis M., Chambon P. and Browns S. Wiley-VCH, Weinheim, 35-86.

Fuchs H., V. Gailus-Durner, T. Adler, J. A. Pimentel, L. Becker et al., 2009 The German Mouse Clinic: a platformvfor systemic phenotype analysis of mouse models. Curr. Pharm. Biotechnol.,10, 236–243.

Fuchs H., V. Gailus-Durner, T. Adler, J. A. Aguilar-Pimentel, L. Becker et al., 2011 Mouse phenotyping. Methods, 53, 120-135.

Gailus-Durner V., H. Fuchs, T. Adler, J. A. Aguilar Pimentel, L. Becker et al., 2009 Systemic first-line phenotyping. Methods Mol. Biol. 530, 463-509.

Hölter S. M., L. Glasl, 2011 High throughput mouse phenotyping. In: Dunnett SB, Lane E [Eds.] Animal models of movement disorders: Vol. I, Neuromethods, 61, Humana Press, 109-133.

Horsch M., S. Schädler, V. Gailus-Durner, H. Fuchs, H. Meyer et al., 2008 Systematic gene expression profiling of mouse model series reveals coexpressed genes. Proteomics, 8, 1248-1256.

Horsch M., P. H. Seeburg, T. Adler, J. A. Aguilar-Pimentel, L. Becker et al., 2011 Requirement of the RNA editing enzyme ADAR2 for normal physiology in mice. JBC 286, 18614-18622.

Lee S., S. Rose, M. D. Metodiev, L. Becker, A. Vernaleken et al., 2015 Overexpression of the mitochondrial methyltransferase TFB1M in the mouse does not impact mitoribosomal methylation status or hearing. Hum Mol Genet. 24, 7286-7294.

Moreth K., R. Fischer, H. Fuchs, V. Gailus-Durner, W. Wurst, et al., 2014 High-throughput phenotypic assessment of cardiac physiology in four commonly used inbred mouse strains. J. Comp. Physiol. B., 184,763-775.

Puk O., C. Dalke, J. Favor, M. Hrabé de Angelis, and J. Graw, 2006 Variations of eye size parameters among different strains of mice. Mamm. Genome, 17, 851-857.

Puk O., M. Hrabé de Angelis, and J. Graw, 2013a Lens density tracking in mice by Scheimpflug imaging. Mamm. Genome, 24, 295-302.

Puk O., M. Hrabě de Angelis, and J. Graw, 2013b Longitudinal fundus and retinal studies with SD-OCT – a comparison of five mouse inbred strains. Mamm. Genome, 24, 198-205.

Prusky G.T., N. M. Alam, S. Beekman, and R. M. Douglas, 2004 Rapid quantification of adult and developing mouse spatial vision using a virtual optomotor system. Invest. Ophthalmol. Vis. Sci., 45, 4611–4616.

Rathkolb B., W. Hans, C. Prehn, H. Fuchs, V. Gailus-Durner et al., 2013a Clinical Chemistry and Other Laboratory Tests on Mouse Plasma or Serum. In Current Protocols in Mouse Biology, Brown S (ed), 3, 69–100.

Rathkolb B., H. Fuchs, V. Gailus-Durner, B. Aigner, E. Wolf, and M. Hrabě de Angelis, 2013b Blood Collection from Mice and Hematological Analyses on Mouse Blood. Curr. Protoc. Mouse Biol., 3, 101-119.

Reinhard C., G. Eder, H. Fuchs, A. Ziesenis, J. Heyder, and H. Schulz, 2002 Inbred strain variation in lung function. Mamm. Genome., 13, 429-37.

Rozman J., M. Klingenspor, and M. Hrabě de Angelis, 2014 A review of standardized metabolic phenotyping of animal models. Mamm. Genome 25, 497-507.

Rozman J., B. Rathkolb, S. Neschen, H. Fuchs, V. Gailus-Durner et al., 2015 Glucose tolerance tests for systematic screening of glucose homeostasis in mice. Curr. Protoc. Mouse Biol., 2, 65-84.

Staropoli J. F., L. Haliw, S. Biswas, L. Garrett, S. M. Hölter et al., 2012 Large-scale phenotyping of an accurate genetic mouse model of JNCL identifies novel early pathology outside the central nervous system. PLoS One. 7, e38310.
