# Supplementary material for: The First Scube3 Mutant Mouse Line with Pleiotropic Phenotypic Alterations
Source: G3 (Bethesda). 2016 Nov 4;6(12):4035–46. doi: 10.1534/g3.116.033670 (PMC5144972; doi:10.1534/g3.116.033670)
Supplement: Supplemental Material [file supp_g3.116.033670_TableS1.pdf]

Supplemental Table 1: Applied phenotyping pipeline in the German Mouse Clinic for the primary phenotype assessment of 15 male and 15 female homozygous *Scube3*$^{N294K/-}$ mutants in comparison to 15 male and 15 female littermate wild-type controls.

**Age (weeks)**	**Research Area**	**Test**	**Reference for the protocol**
9	Behavior	Open Field test	Hölter and Glasl (2011)
9	Neurology	Modified SHIRPA, grip strength	Gailus-Durner et al. (2009)
10	Behavior	Acoustic startle response and pre-pulse inhibition	Horsch et al. (2011)
10	Neurology	Rotarod	Gailus-Durner et al. (2009)
11	Clinical Chemistry	Fasting plasma lipid and glucose values	Rathkolb et al. (2013a)
12	Nociception	Hot plate test	Fuchs et al. (2009)
12	Bone and Cartilage	Morphological observation	Fuchs et al. (2000)
13	Metabolism	Indirect calorimetry and NMR body composition	Rozman et al. (2014)
14	Diabetes	Intraperitoneal Glucose	Rozman et al.

		tolerance test	(2015)
15	Cardiovascular	Awake electrocardiogram and echocardiography	Moreth et al. (2014)
16	Eye and vision	Scheimpflug imaging, OCT, laser interference biometry, optokinetic drum	Puk et al. (2013a), Puk et al. (2013b), Puk et al. (2006), Prusky et al. (2004)
17	Clinical chemistry	Clinical chemistry (ad lib. fed)	Rathkolb et al. (2013a)
17	Clinical chemistry	Hematology	Rathkolb et al. (2013b)
17	Immunology	FACS analysis of peripheral blood cells and immunoglobulins	Fulton et al. (1997), Fuchs et al. (2011)
17	Steroid-biochemistry	Analysis of corticosterone, testosterone and androstenedione	Rathkolb et al. (2013a)
18	Neurology	Auditory brainstem response	Lee et al. (2015)
18	Bone and cartilage	X-ray analysis, bone densitometry by DEXA	Fuchs et al. (2006)
19	Metabolism	NMR body composition	Rozman et al. (2014)
21	Lung function	Lung function analysis	Reinhard et al.

			(2002)
21	Transcriptome analysis	Tissue collection, and expression profiling of kidney samples	Horsch et al. (2008)
21	Pathology	Macro- and microscopic analysis (3 mice per genotype and sex)	Staropoli et al. (2012)
34	Clinical Chemistry	Renal function test	Fuchs et al. (2011)
