# Supplementary material for: The First Scube3 Mutant Mouse Line with Pleiotropic Phenotypic Alterations
Source: G3 (Bethesda). 2016 Nov 4;6(12):4035–46. doi: 10.1534/g3.116.033670 (PMC5144972; doi:10.1534/g3.116.033670)
Supplement: Supplemental Material [file supp_g3.116.033670_TableS2.pdf]

Supplemental Table 2: Parameters that showed relevant and significant changes in primary GMC phenotypic analysis pipeline

A) Parameters analyzed by Wilcoxon test

**Test**	**Parameter**	**Scube3^{wt} female**	***Scube3$^{N294K/-}$* female**	**Scube3^{wt} male**	***Scube3$^{N294K/-}$* male**	**female**	**male**	**overall**
		**n=9**	**n=11**	**n=10**	**n=10**			
Auditory brainstem response	Click ABR [dB]	30 [30 , 30]	35 [35 , 35]	30 [30 , 30]	35 [35 , 35]	0.011	0.003	< 0.001
	Threshold at 6 kHz [dB]	40 [40 , 45]	50 [48 , 55]	48 [45 , 50]	50 [46 , 55]	0.004	0.213	0.002
	Threshold at 12 kHz [dB]	25 [25 , 30]	30 [25 , 38]	25 [25 , 30]	30 [30 , 35]	0.207	0.146	0.03
	Threshold at 18 kHz [dB]	30 [25 , 30]	35 [30 , 42]	30 [26 , 35]	35 [35 , 35]	0.021	0.054	0.001
	Threshold at 24 kHz [dB]	35 [30 , 35]	40 [38 , 48]	35 [35 , 39]	38 [35 , 40]	0.013	0.332	0.005

	Threshold at 30 kHz [dB]	45 [40 , 45]	50 [48 , 60]	45 [45 , 50]	50 [50 , 54]	0.028	0.108	0.004
		**n=6**	**n=8**	**n=6**	**n=8**			
Cardiology	QRS interval duration [ms]	9.5 [8.2 , 10.8]	9.5 [8.8 , 10.2]	8.5 [8 , 9]	9 [9 , 9.2]	0.956	0.028	0.215
	Interventricular septum width in systole [mm]	0.54 [0.51 , 0.54]	0.48 [0.47 , 0.52]	0.54 [0.5 , 0.57]	0.51 [0.48 , 0.54]	0.01	0.072	0.003
	Interventricular septum width in diastole [mm]	0.55 [0.5 , 0.57]	0.54 [0.5 , 0.56]	0.55 [0.51 , 0.57]	0.5 [0.47 , 0.52]	0.55	0.01	0.022
	Left ventricular posterior wall width in systole [mm]	0.62 [0.58 , 0.65]	0.62 [0.57 , 0.65]	0.66 [0.64 , 0.69]	0.63 [0.58 , 0.64]	0.755	0.01	0.138
	Respiration Rate [1/min]	748.05 [724.53 , 764.95]	716.42 [642.68 , 730.96]	727.27 [695.72 , 746.2]	742.27 [725.45 , 755.91]	0.027	0.289	0.388
	Left ventricular mass [mg]	24.96 [22.87 , 26.11]	20.8 [18.29 , 24.33]	27.9 [23.24 , 33.76]	21.18 [19.35 , 24.87]	0.155	0.007	0.002
		**n=9**	**n=9**	**n=9**	**n=9**			
	Heart weight [mg]	131 [127 , 133]	106 [104 , 114]	162 [158 , 164]	117 [113 , 122]	0.001	0.001	0.001
	Tibia length [mm]	19.11 [19.08 , 19.39]	18.13 [17.9 , 18.17]	19.03 [18.98 , 19.43]	17.81 [17.76 , 18.48]	0.001	0.001	0.001
	Heart weight / Tibia length [mg/mm]	6.8 [6.64 , 6.97]	5.92 [5.74 , 6.27]	8.3 [8.16 , 8.44]	6.5 [6.33 , 6.63]	0.004	0.001	0.001
	Heart Weight / Body Weight [mg/g]	3.84 [3.7 , 3.95]	4.74 [4.55 , 5.26]	4.12 [3.74 , 4.19]	4.58 [4.43 , 4.86]	0.001	0.001	0.001
		**n=14**	**n=14**	**n=15**	**n=15**			
Immunology	Ig G1	180.97 [145.48 , 249.18]	302.11 [242.04 , 347.65]	272.43 [223.28 , 323.6]	231.12 [205.9 , 283.41]	0.005	0.561	0.104
	Ig G3	1292.57 [980.27 , 1447.29]	618.04 [477.85 , 724.58]	1027.16 [777.23 , 1234.4]	757.24 [703.42 , 857.62]	0.001	0.023	< 0.001

	CD3+CD8+/CD62L+	87.2 [81.42 , 92.62]	78 [69.6 , 85.95]	87.9 [84.8 , 89.85]	81.3 [79.2 , 87.15]	0.032	0.054	0.003
	NK+/CD11b+	83.7 [82.05 , 84.33]	85.25 [84.72 , 85.83]	71.2 [69.85 , 73.2]	75.1 [72.45 , 75.6]	0.002	0.026	0.066
		**n=14**	**n=14**	**n=15**	**n=15**			
Steroids	Corticosterone [nmol/l]	321.83 [238.48 , 386.77]	286.18 [238.48 , 321.1]	154.71 [122.38 , 188.62]	121.52 [102.18 , 143.88]	0.223	0.021	0.181

B) Parameters analyzed by linear model with sex and genotype as predictors

**Test**	**Parameter**	**Scube3^{wt} female**	***Scube3$^{N294K/-}$*** **female**	**Scube3^{wt} male**	***Scube3$^{N294K/-}$*** **male**	**genotype**	**sex**	**sex: genotype**
		**n=14**	**n=13**	**n=15**	**n=15**			
DEXA	Bone mineral density [mg/cm²]	58 ± 3	51 ± 4	60 ± 3	49 ± 3	< 0.001	0.513	0.043
	Bone mineral content [mg]	613 ± 97	378 ± 60	765 ± 174	410 ± 60	< 0.001	0.004	0.043
	Body length [cm]	10.01 ± 0.17	9.03 ± 0.28	10.22 ± 0.24	8.98 ± 0.16	< 0.001	0.306	0.029
		**n=14**	**n=16**	**n=15**	**n=15**			
Clinical-chemistry after fasting	Glucose [mmol/l]	11.12 ± 2.18	9.01 ± 1.47	10.88 ± 3.26	9.18 ± 2.41	0.003	0.955	0.743
	Triglycerides [mmol/l]	2.299 ± 0.744	1.012 ± 0.336	2.045 ± 0.299	1.32 ± 0.36	< 0.001	0.822	0.022
	NEFA [mmol/l]	1.15 ± 0.21	0.88 ± 0.21	0.83 ± 0.2	0.74 ± 0.17	0.001	< 0.001	0.085
	Glycerol [mmol/l]	0.335 ± 0.062	0.263 ± 0.067	0.25 ± 0.062	0.226 ± 0.06	0.005	< 0.001	0.144
		**n=14**	**n=15**	**n=15**	**n=15**			
Clinical-chelimstry	Calcium [mmol/l]	2.4 ±0.03	2.48 ±0.11	2.45 ±0.04	2.45 ±0.04	0.024	0.452	0.018

(nonfasted)	Inorganic phosphate [mmol/l]	1.57 ±0.31	1.34 ±0.21	1.67 ±0.19	1.57 ±0.15	0.005	0.006	0.272
	ALP [µmol/l]	136 ±12	142 ±13	93 ±7	101 ±6	0.014	< 0.001	0.683
	Cholesterol [mmol/l]	2.905 ± 0.251	2.93 ± 0.224	4.655 ± 0.29	4.227 ± 0.264	0.005	< 0.001	0.002
	Triglyceride [mmol/l]	3.453 ± 0.594	2.721 ± 0.812	3.632 ± 0.784	2.662 ± 0.525	< 0.001	0.743	0.516
	alpha-Amylase [U/l]	539.78 ± 28.57	598.71 ± 34.07	601.74 ± 30.36	653.67 ± 29.04	< 0.001	< 0.001	0.664
	Glucose [mmol/l]	9.67 ± 1.06	9.36 ± 1	11.17 ± 0.91	10.39 ± 0.87	0.034	< 0.001	0.364
	Potassium [mmol/l]	4 ± 0.2	4.3 ± 0.3	4.6 ± 0.2	4.6 ± 0.3	0.01	< 0.001	0.046
	Total protein [g/l]	54.9 ± 1.6	55 ± 1.9	59.2 ± 1.3	56.2 ± 1.3	0.001	<0.001	< 0.001
	Albumin [g/l]	26.8 ± 0.8	26.9 ± 0.9	26.5 ± 0.6	25.1 ± 0.6	0.001	< 0.001	< 0.001
	Creatinine enz. [mumol/l]	13.92 ± 2.2	16.98 ± 2.49	14.88 ± 1.66	16.81 ± 1.64	< 0.001	0.462	0.296
	Urea [mmol/l]	10.46 ± 1.23	13.55 ± 1.71	9.27 ± 0.73	12.4 ± 0.64	< 0.001	< 0.001	0.94
		**n=14**	**n=15**	**n=15**	**n=15**			
Glucose tolarance test	Glucose (T=0)	4.72 ± 0.68	3.9 ± 1.35	5.69 ± 1.14	5.06 ± 0.45	0.006	< 0.001	0.71
	AUC 0-30 min.	183.64 ± 26.04	149.09 ± 54.7	235.27 ± 43.53	191.92 ± 28.31	< 0.001	< 0.001	0.675
	AUC 30-120 min.	373.89 ± 87.27	220.76 ± 116.26	599.84 ± 133.64	342.18 ± 81.26	< 0.001	< 0.001	0.066
		**n=14**	**n=16**	**n=15**	**n=15**			
Open field	Distance traveled	9551.54 ±1761.41	6954.86 ±2393.69	9138.58 ±2260.13	6747.58 ±2190.89	< 0.001	0.583	0.856
	Number of rears	37.36 ±40.45	8.56 ±14.08	60.6 ±45.99	16.07 ±28.25	< 0.001	0.086	0.375
	Percent distance in the center	19.57 ±14.47	10.1 ±11.82	22.05 ±14.72	11.25 ±14.53	0.007	0.615	0.854

	Percent time spent in the center	15.07 ±13.86	7.43 ±9.85	17.77 ±12.62	9.54 ±13.13	0.016	0.456	0.927
	Whole arena - average speed	8.36 ±1.78	5.88 ±2.09	8.13 ±2.26	5.79 ±2.03	< 0.001	0.77	0.889
	Latency to enter in the center	60.32 ±71	285.08 ±396.33	81.2 ±96.51	344.33 ±398.97	0.002	0.598	0.8
	Number of entries in the center	97.86 ±51.91	53.38 ±61.5	107.33 ±76.46	45.6 ±46.1	0.001	0.957	0.582
	Whole arena distance	9551.54 ±1761.41	6954.86 ±2393.69	9138.58 ±2260.13	6747.58 ±2190.89	< 0.001	0.583	0.856
		**n=5**	**n=10**	**n=11**	**n=12**			
Modified hole board	Risk assessment [latency]	129.06 ± 69.81	195.92 ± 42.73	250.62 ± 33.14	118.96 ± 32.94	0.455	0.606	0.027
	Maximum velocity [cm/sec]	56 ± 5.39	49.81 ± 4.68	59.98 ± 3.96	44.43 ± 2	0.012	0.866	0.261
	Angular velocity [degrees/sec]	240.33 ± 28.76	177.04 ± 13.95	250.42 ± 31.73	184.09 ± 9.27	0.009	0.718	0.951
		**n=14**	**n=16**	**n=15**	**n=15**			
SHIRPA	Locomotor Activity (SHIRPA)	7.14 ± 5.35	4.56 ± 5.35	9.13 ± 6.49	6 ± 5.44	0.057	0.248	0.851

C) Parameters analyzed by Linear model with sex, genotype and body weight as predictors (in case of femur length, body length was used instead of body weight)

Test	parameter	Scube3^wt female	*Scube3^N294K/-* female	Scube3^wt male	*Scube3^N294K/-* male	genotype	sex	weight	sex:weight	genotype:weight	genotype:sex	genotype:sex:weight
		**n=14**	**n=16**	**n=15**	**n=15**							
Grip Strength	mean grip strength (4 paws)	203.87± 7.61	149.22± 16.7	206.18± 15.45	178.28± 21.11	0.01	0.71	<0.001	0.47	0.02	0.11	0.11
		**n=14**	**n=15**	**n=15**	**n=15**							
NMR Body composition	Body mass (g)	25.5 ± 1.8	18.5 ± 1.6	31 ± 2.3	22.1 ± 1.2	<0.001	<0.001	NA	NA	NA	0.031	NA
	Fat mass (g)	6.1 ± 1.2	3.5 ± 0.6	7.2 ± 1.1	4.1 ± 0.4	<0.001	<0.001	<0.001	NA	NA	0.024	NA
	Lean mass (g)	15.6 ± 0.7	12 ± 0.9	19.3 ± 1.1	14.5 ± 0.6	<0.001	0.003	<0.001	NA	NA	0.02	NA
		**n=14**	**n=15**	**n=15**	**n=15**							
Indirect calorimetry	avg. VO2 [ml/(h animal)]	88.821 ± 11.76	70.833 ± 9.415	87.663 ± 5.948	70.518 ± 5.499	<0.001	0.454	<0.001	NA	NA	0.157	NA
	min. VO2 [ml/(h animal)]	59.214 ± 9.283	46.333 ± 11.101	59.933 ± 6.273	44.267 ± 7.851	0.002	0.377	<0.001	NA	NA	0.73	NA
	max. VO2 [ml/(h animal)]	133.357 ± 12.524	106.933 ± 12.959	135.4 ± 11.855	111.8 ± 6.951	0.049	0.761	0.001	NA	NA	0.159	NA
	avg. distance [cm]	6662 ± 4157	6384 ± 4536	3763 ± 1313	3430 ± 1725	0.001	0.719	NA	NA	NA	0.974	NA

	avg. rearing [counts]	157 ± 102	85 ± 59	109 ± 45	59 ± 24	0.03	0.001	NA	NA	NA	0.512	NA
		**n=14**	**n=15**	**n=14**	**n=15**							
X-ray analysis	Femur length [mm]	15.47 ± 0.34	13.23 ± 0.33	14.63 ± 0.42	12.70 ± 0.34	<0.001	<0.001	0.133	NA	NA	0.086	NA
