# Supplementary material for: The First Scube3 Mutant Mouse Line with Pleiotropic Phenotypic Alterations
Source: G3 (Bethesda). 2016 Nov 4;6(12):4035–46. doi: 10.1534/g3.116.033670 (PMC5144972; doi:10.1534/g3.116.033670)
Supplement: Supplemental Material [file supp_g3.116.033670_TableS3.pdf]

Supplemental Table 3: pQCT analysis of 9 and 12 months old *Scube3* $^{N294K/-}$ mice

| **Age** | **Analyzed Area** | **Parameter** | ***Scube3*$^{WT}$ female** | ***Scube3*$^{N294K/-}$ female** | ***Scube3*$^{WT}$ male** | ***Scube3*$^{N294K/-}$ e** | **genotype** | **sex** | **sex: genotype** |
|---|---|---|---|---|---|---|---|---|---|
| | | | **n=10** | **n=11** | **n=10** | **n=7** | | | |
| 9 months | femoral metaphysis | Total content [mg] | 2.93 ± 0.26 | 2.66 ± 0.4 | 2.38 ± 0.15 | 2.2 ± 0.32 | 0.031 | < 0.001 | 0.646 |
| 9 months | femoral metaphysis | Cortical / subcortical area [mm^2] | 2.97 ± 0.33 | 2.6 ± 0.33 | 2.37 ± 0.19 | 2.19 ± 0.39 | 0.01 | < 0.001 | 0.384 |
| 9 months | femoral metaphysis | Cortical area [mm^2] | 2.82 ± 0.32 | 2.47 ± 0.33 | 2.23 ± 0.17 | 2.07 ± 0.38 | 0.015 | < 0.001 | 0.355 |
| 9 months | femoral metaphysis | Trabecular density [mm^2] | 283.6 ± 22.5 | 263.5 ± 37.8 | 293.4 ± 28.9 | 259.6 ± 31 | 0.012 | 0.772 | 0.501 |
| 9 months | femoral metaphysis | Cortical thickness c [mm] | 0.573 ± 0.057 | 0.498 ± 0.083 | 0.386 ± 0.033 | 0.371 ± 0.089 | 0.048 | < 0.001 | 0.191 |
| 9 months | femoral diaphysis | Total content [mg] | 2.8 ± 0.22 | 2.33 ± 0.26 | 2.56 ± 0.21 | 2.38 ± 0.19 | < 0.001 | 0.235 | 0.059 |

| | | | | | | | | | |
|---|---|---|---|---|---|---|---|---|---|
| 9 months | femoral diaphysis | Total density [mg/cm^3] | 1172.12 ± 35.26 | 1010.01 ± 69.83 | 1005.56 ± 43.33 | 972.14 ± 91.84 | < 0.001 | < 0.001 | 0.003 |
| 9 months | femoral diaphysis | Cortical / subcortical content [mg] | 2.75 ± 0.22 | 2.26 ± 0.27 | 2.48 ± 0.22 | 2.3 ± 0.2 | < 0.001 | 0.128 | 0.05 |
| 9 months | femoral diaphysis | Cortical / subcortical density [mg/cm^3] | 1248.23 ± 26.45 | 1161.92 ± 50.79 | 1126.35 ± 30.23 | 1109.37 ± 48.68 | < 0.001 | < 0.001 | 0.013 |
| 9 months | femoral diaphysis | Cortical / subcortical area [mm^2] | 2.2 ± 0.16 | 1.94 ± 0.17 | 2.2 ± 0.17 | 2.07 ± 0.15 | 0.001 | 0.261 | 0.239 |
| 9 months | femoral diaphysis | Cortical content [mg] | 2.71 ± 0.22 | 2.22 ± 0.27 | 2.43 ± 0.21 | 2.25 ± 0.2 | < 0.001 | 0.108 | 0.051 |
| 9 months | femoral diaphysis | Cortical density [mg/cm^3] | 1289.4 ± 24.1 | 1206.4 ± 49.4 | 1165.1 ± 26.1 | 1153.3 ± 53.1 | 0.001 | < 0.001 | 0.01 |
| 9 months | femoral diaphysis | Cortical area [mm^2] | 2.1 ± 0.15 | 1.84 ± 0.17 | 2.09 ± 0.17 | 1.95 ± 0.15 | 0.001 | 0.348 | 0.226 |
| 9 months | femoral diaphysis | Trabecular area [mm^2] | 0.18 ± 0.04 | 0.36 ± 0.07 | 0.35 ± 0.06 | 0.4 ± 0.19 | 0.001 | 0.003 | 0.049 |
| 9 months | femoral diaphysis | Endosteal circumference c [mm] | 1.875 ± 0.171 | 2.411 ± 0.207 | 2.392 ± 0.182 | 2.512 ± 0.425 | < 0.001 | 0.001 | 0.015 |
| 9 months | femoral diaphysis | Cortical thickness c [mm] | 0.573 ± 0.032 | 0.472 ± 0.039 | 0.519 ± 0.032 | 0.485 ± 0.048 | < 0.001 | 0.114 | 0.01 |
| | | | n=11 | n=7 | n=10 | n=7 | | | |
| 12 months | femoral metaphysis | Total content [mg] | 3.05 ± 0.23 | 3.06 ± 0.45 | 2.32 ± 0.25 | 2.28 ± 0.27 | 0.916 | < 0.001 | 0.832 |

| | | | | | | | | | |
|---|---|---|---|---|---|---|---|---|---|
| 12 months | femoral metaphysis | Total density [mg/cm^3] | 807.37 ± 51.36 | 802.05 ± 81.87 | 585.66 ± 54.69 | 580.34 ± 50.35 | 0.796 | < 0.001 | 1 |
| 12 months | femoral metaphysis | Total area [mm^2] | 3.78 ± 0.25 | 3.82 ± 0.45 | 3.95 ± 0.15 | 3.93 ± 0.28 | 0.929 | 0.156 | 0.784 |
| 12 months | femoral metaphysis | Cortical / subcortical content [mg] | 2.87 ± 0.28 | 2.87 ± 0.48 | 1.84 ± 0.29 | 1.82 ± 0.32 | 0.938 | < 0.001 | 0.95 |
| 12 months | femoral metaphysis | Cortical / subcortical density [mg/cm^3] | 909.16 ± 27.67 | 936.24 ± 49.28 | 799.42 ± 44.22 | 798.98 ± 32.36 | 0.325 | < 0.001 | 0.309 |
| 12 months | femoral metaphysis | Cortical / subcortical area [mm^2] | 3.16 ± 0.32 | 3.08 ± 0.55 | 2.3 ± 0.31 | 2.28 ± 0.34 | 0.675 | < 0.001 | 0.814 |
| 12 months | femoral metaphysis | Cortical content [mg] | 2.81 ± 0.27 | 2.82 ± 0.47 | 1.79 ± 0.29 | 1.77 ± 0.31 | 0.947 | < 0.001 | 0.914 |
| 12 months | femoral metaphysis | Cortical density [mg/cm^3] | 933.8 ± 29 | 959.5 ± 49.2 | 824.5 ± 45.6 | 825.7 ± 32.7 | 0.331 | < 0.001 | 0.374 |
| 12 months | femoral metaphysis | Cortical area [mm^2] | 3.02 ± 0.31 | 2.95 ± 0.52 | 2.17 ± 0.31 | 2.14 ± 0.34 | 0.692 | < 0.001 | 0.871 |

| | | | | | | | | | |
|---|---|---|---|---|---|---|---|---|---|
| 12 months | femoral metaphysis | Trabecular content [mg] | 0.18 ± 0.07 | 0.19 ± 0.07 | 0.47 ± 0.06 | 0.46 ± 0.09 | 0.99 | < 0.001 | 0.591 |
| 12 months | femoral metaphysis | Trabecular density [mm^2] | 287.9 ± 28.9 | 263.5 ± 33.5 | 288.7 ± 28 | 277.8 ± 28.6 | 0.093 | 0.468 | 0.513 |
| 12 months | femoral metaphysis | Trabecular area [mm^2] | 0.62 ± 0.25 | 0.74 ± 0.31 | 1.65 ± 0.24 | 1.65 ± 0.3 | 0.51 | < 0.001 | 0.514 |
| 12 months | femoral metaphysis | Periosteal circumference c [mm] | 6.889 ± 0.226 | 6.915 ± 0.405 | 7.046 ± 0.137 | 7.026 ± 0.262 | 0.972 | 0.143 | 0.799 |
| 12 months | femoral metaphysis | Endosteal circumference c [mm] | 3.059 ± 0.495 | 3.252 ± 0.631 | 4.729 ± 0.308 | 4.738 ± 0.383 | 0.531 | < 0.001 | 0.566 |
| 12 months | femoral metaphysis | Periosteal circumference [mm] | 6.691 ± 0.313 | 6.385 ± 1.528 | 6.87 ± 0.241 | 6.715 ± 0.244 | 0.357 | 0.311 | 0.762 |
| 12 months | femoral metaphysis | Endosteal circumference [mm] | 0.983 ± 1.197 | 1.03 ± 1.398 | 2.268 ± 2.222 | 3.59 ± 2.024 | 0.266 | 0.003 | 0.3 |
| 12 months | femoral metaphysis | Cortical thickness c [mm] | 0.61 ± 0.081 | 0.583 ± 0.114 | 0.369 ± 0.058 | 0.364 ± 0.061 | 0.579 | < 0.001 | 0.687 |
| 12 months | femoral metaphysis | Cortical thickness [mm] | 0.26 ± 0.275 | 0.233 ± 0.191 | 0.16 ± 0.159 | 0.302 ± 0.162 | 0.432 | 0.83 | 0.251 |

| | | | | | | | | | |
|---|---|---|---|---|---|---|---|---|---|
| 12 months | femoral metaphysis | Cortical thickness SD [mm] | 0.0759 ± 0.0801 | 0.0963 ± 0.0776 | 0.066 ± 0.0694 | 0.1183 ± 0.0625 | 0.161 | 0.814 | 0.533 |
| 12 months | femoral diaphysis | Total content [mg] | 2.95 ± 0.19 | 2.79 ± 0.27 | 2.55 ± 0.31 | 2.32 ± 0.31 | 0.046 | < 0.001 | 0.711 |
| 12 months | femoral diaphysis | Total density [mg/cm^3] | 1166.16 ± 55.66 | 1025.49 ± 43.73 | 966.04 ± 84.98 | 871.74 ± 79.01 | < 0.001 | < 0.001 | 0.333 |
| 12 months | femoral diaphysis | Cortical / subcortical content [mg] | 2.91 ± 0.19 | 2.72 ± 0.26 | 2.45 ± 0.32 | 2.2 ± 0.31 | 0.024 | < 0.001 | 0.752 |
| 12 months | femoral diaphysis | Cortical / subcortical density [mg/cm^3] | 1238.68 ± 31.84 | 1164.31 ± 30.93 | 1102.6 ± 58.62 | 1035.99 ± 58.31 | < 0.001 | < 0.001 | 0.811 |
| 12 months | femoral diaphysis | Cortical content [mg] | 2.86 ± 0.19 | 2.67 ± 0.26 | 2.4 ± 0.32 | 2.15 ± 0.31 | 0.023 | < 0.001 | 0.771 |
| 12 months | femoral diaphysis | Cortical density [mg/cm^3] | 1280.5 ± 29.4 | 1207 ± 27.6 | 1141.5 ± 58.8 | 1072.8 ± 57.6 | < 0.001 | < 0.001 | 0.881 |
| 12 months | femoral diaphysis | Endosteal circumference c [mm] | 1.899 ± 0.328 | 2.53 ± 0.238 | 2.58 ± 0.24 | 2.866 ± 0.339 | < 0.001 | < 0.001 | 0.097 |
| 12 months | femoral diaphysis | Cortical thickness c [mm] | 0.595 ± 0.051 | 0.528 ± 0.037 | 0.505 ± 0.049 | 0.463 ± 0.043 | 0.002 | < 0.001 | 0.426 |
